# Direct-Write Fabrication of Cellulose Nano-Structures via Focused Electron Beam Induced Nanosynthesis

**DOI:** 10.1038/srep32451

**Published:** 2016-09-02

**Authors:** Thomas Ganner, Jürgen Sattelkow, Bernhard Rumpf, Manuel Eibinger, David Reishofer, Robert Winkler, Bernd Nidetzky, Stefan Spirk, Harald Plank

**Affiliations:** 1 Institute for Electron Microscopy and Nanoanalysis, Graz University of Technology, Steyrergasse 17, A-8010 Graz, Austria; 2 Institute of Biotechnology and Biochemical Engineering, Graz University of Technology, Petersgasse 12, A-8010 Graz, Austria; 3 Institute for Chemistry and Technology of Materials, Graz University of Technology, Stremayrgasse 9, 8010 Graz, Austria; 4 Graz Centre for Electron Microscopy, Steyrergasse 17, A-8010 Graz, Austria; 5 Austrian Centre of Industrial Biotechnology, Petersgasse 14, A-8010 Graz, Austria

## Abstract

In many areas of science and technology, patterned films and surfaces play a key role in engineering and development of advanced materials. Here, we introduce a new generic technique for the fabrication of polysaccharide nano-structures via focused electron beam induced conversion (FEBIC). For the proof of principle, organosoluble trimethylsilyl-cellulose (TMSC) thin films have been deposited by spin coating on SiO_2_ / Si and exposed to a nano-sized electron beam. It turns out that in the exposed areas an electron induced desilylation reaction takes place converting soluble TMSC to rather insoluble cellulose. After removal of the unexposed TMSC areas, structured cellulose patterns remain on the surface with FWHM line widths down to 70 nm. Systematic FEBIC parameter sweeps reveal a generally electron dose dependent behavior with three working regimes: incomplete conversion, ideal doses and over exposure. Direct (FT-IR) and indirect chemical analyses (enzymatic degradation) confirmed the cellulosic character of ideally converted areas. These investigations are complemented by a theoretical model which suggests a two-step reaction process by means of *TMSC* → *cellulose* and *cellulose* → *non-cellulose material* conversion in excellent agreement with experimental data. The extracted, individual reaction rates allowed the derivation of design rules for FEBIC parameters towards highest conversion efficiencies and highest lateral resolution.

Polysaccharides are a large class of biopolymers which exhibit a large structural and chemical diversity and, consequently, a variety of biological functions^1^. Among all polysaccharides, cellulose, a homopolymer of β-(1,4) linked D-glucose units, is of particular importance from both academic and industrial point of view. It is highly abundant since it is a major constituent of higher plant cell walls and some bacteria. Further, it offers a wide range of applications in many areas ranging from packaging, textiles, papers, housing to medicine, life sciences as well as advanced materials to mention just some examples^2^,^3^,^4^,^5^,^6^,^7^. In this context, cellulose nanomaterials such as nanofibrils, nanocrystals, aerogels or thin films have seen a tremendous rise during the past years, since it allowed materials scientists to shift polysaccharide materials from the micro- to the nanoscale world concomitant with new application areas of cellulosic materials^2^. However, for many applications (e.g. in electronics) thin films featuring well-defined patterns in the nanometer regime are required which are hardly realized so far for polysaccharides in general and cellulose in particular. A major problem in the processing of cellulose for this purpose is its poor solubility in common organic solvents. However, the use of soluble derivatives such as organosoluble trimethylsilyl cellulose (**TMSC**), which is converted to cellulose after the processing step via acid vapor hydrolysis, allows for a facile preparation of cellulose thin films. Introduced by Klemm and further developed by Kontturi, this method provides amorphous thin films with a flat and defined morphology in combination with easily adjustable film thickness ranging from a few nanometers to several micrometers^8^,^9^. Film properties have been exploited in numerous studies to elucidate the basic interaction principles of cellulose with other biomolecules such as proteins, DNA and other polysaccharides but also allowed for investigations into the interaction of cellulose with water^10^,^11^,^12^,^13^,^14^,^15^,^16^,^17^,^18^,^19^,^20^,^21^. However, in order to provide a convenient platform for integration in electronics, bio-sensing or diagnostic applications another key demand must be met: the defined lateral pre-structuring of cellulose on the macro-, micro- and nanoscale. First protocols were demonstrated by Tanaka *et al.*^22^ who employed UV etching of regenerated cellulose films to create macro and micrometer sized pads as protein supports. However, a drawback of this approach is its intrinsic destructive nature creating defects at the edges of the patterns^22^, which negatively impacts the performance at very small feature sizes. Later, Spirk *et al.* and Werner *et al*.^23^ reported macrostructured cellulose pads derived from TMSC^24^,^25^. The patterning was achieved by applying a metal mask having holes onto the TMSC films during the acid vapor hydrolysis^24^,^25^ or by using a novel lift-off technique to remove specific cellulose areas with a PEI coated cation stamp^23^. Cellulose microstructures using a combination of soft lithography and enzymes have been realized by Kargl *et al.* who used a microstructured mold having micrometer sized channels in combination with enzymes^25^. By pressing the mold onto the cellulose thin film and subsequent deposition of cellulose digesting enzymes micrometer patterns were obtained.^25^ However, a major drawback is that large areas are difficult to pattern and further the procedure is rather laborious and difficult to upscale. To overcome this issue, an impressive study^26^ was presented using photocatalytic regeneratoin of a TMSC/N-hydroxynaphtalimide triflate (**NHNA**) blend. At wavelengths higher than 300 nm (**UV**) photolysis of NHNA yields triflic acid. Thereby, the acidic proton performs nucleophilic attack of the TMS-O bond and leads to re-substitution to cellulose. Photo-regeneration proved to be a feasible method to obtain structures in the micrometer range and below. Additionally, two-photon lithography (**TPA**) was demonstrated at the same system which resulted in feature sizes of approx. 600 nm. Although simple in principle, the large feature size and traces of remaining NHNA might be detrimental for specific applications. Another approach to produce larger patterns within short times was introduced by Taajamaa *et al.*^27^ by using a polysaccharid/polysterene blend. Although this technique allows fast and large scale structuring it slightly lacks lateral position control and size fidelity. In particular, applications in microelectronics^28^,^29^,^30^, sensors^31^,^32^,^33^,^34^,^35^ and nanofluidics^36^ require the possibility to generate nanostructures below 100 nm. A recent study by Taskei *et al.*^37^ which used a different cellulose based resist material showed that electron lithography on cellulose-derivates is a feasible method to fabricate, e.g., nanostructured masks for semiconductor industry and demonstrates the significance for industry.

Based on this motivation, we here demonstrate a highly localized, direct conversion of TMSC layers into cellulose via a nano-sized focused electron beam as used in classical scanning electron microscopes (**SEM**). The conversion effect resembles the basic principle of e-beam lithography where the electron beam chemically changes a thin photo-resist^38^,^39^,^40^. Depending on the resist type (positive or negative), the exposed areas are removed or remain on the surface via a wet chemical process. In our approach the focused electron beam directly transfers TMSC into cellulose. After removal of the unexposed regions via a final wet-chemical process, cellulose structures remain on the surface with features sizes below 100 nm. The study first focuses on the proof-of-principle by 1) using cellulose specific enzymes and atomic force microscopy (**AFM**) to quantitatively access converted cellulose; and 2) apply Fourier Transform Infrared (**FT-IR**) spectroscopy to gain more detailed chemical information of ideally converted regions. Next, a detailed parameter sweep during fabrication is presented which reveals three different regimes during conversion: ***1)*** incompletely converted, ***2)*** ideally converted, and ***3)*** over exposed. The gathered data is then combined with a theoretical model which explains the observed regimes and allow determination of ideal process parameters for efficient and chemically ideal conversion. The final part focuses on the downscaling which reveals that this method is indeed capable to produce cellulose structures in the sub−100 nm regime via this direct write conversion approach.

## Results and Discussion

### Preliminary Experiments

During the last decade direct-write nanofabrication via focused electron beam induced deposition (**FEBID**) has attracted considerable attention^41^,^42^,^43^. This technology uses gaseous precursor molecules which absorb on practically any given surface in a classical SEM vacuum chamber. The interaction between these molecules and the focused electron beam leads to a highly localized chemical dissociation and immobilization which forms the functional deposit with spatial nanometer resolution. Similar in principle, electron beam lithography uses electron sensitive resists like poly(methyl methacrylate) (PMMA)^44^ to achieve a structuring mask for a later development process. As a positive resist, electron irradiation causes degradation of PMMA in fragments of low molecular weight. It is conceivable that similar processes may be used to regenerate TMSC to cellulose. Traditionally, TMSC regeneration is achieved by use of acidic or basic reagents which catalyze the de-silylation of TMSC into cellulose via hydrophilic attack at the central silicon atom. During this process, volatile trimethylsilanol (TMSiOH) and hexamethyldisiloxane (TMSi_2_O) are formed which can leave the films as suggested by Kontturi *et al.*^7^,^45^. On films, this reaction has been readily explored using a variety of techniques, either *in-situ* (QCM-D, GI-SAXS) or *ex-situ* (XRR, ATR-IR, wettability measurements, XPS)^9^,^17^,^46^,^47^,^48^. In the case of electron induced regeneration, the process still requires nucleophilic attack at the central silicon atom which may state a bottleneck as the reactive species have to be generated within the film. To bypass this problem we initially used a humid low vacuum atmosphere for preliminary experiments. The interaction of the beam and the water molecules may lead to dissociation and nucleophilic attack similar to the acidic reagents. For completeness we performed the same experiments in a high vacuum and thus water free environment (detailed results can be found in supplement 1). In brief, we achieved a contrary result to the proposed and hypothesized better regeneration under humid low vacuum atmosphere. We showed that using low vacuum conditions including water is rather detrimental to the process which primarily is caused by the so called curtaining effect. On the other hand, high vacuum conditions revealed that TMSC may be regenerated only by the interaction with the electron beam. A significant change in film height seen as a change of interference color is visible for the high vacuum patterns after application of cellulases (see supplement 1). We so far can only speculate about the exact mechanisms of the generation of the protons required for the nucleophilic attack but it seems likely that the nucleophile is provided from the TMS moiety itself after cleavage. As proposed by Royall *et al.*^49^ for water, electron beam interaction with in particular organic matter produces a large number of reactive compounds including protons needed for regeneration. As the TMS moiety is rich in hydrogen this process seems to be the likely cause of the regeneration in high vacuum. Despite the necessity to get a clearer understanding of process associated chemistry, we first have to unravel the relevant process parameters. From here on, we denote the process as Focused Electron Beam Induced Conversion (**FEBIC**) and provide a detailed process parameter study and its conversion implications in the following.

### Parameter Space

**Figure 1:**
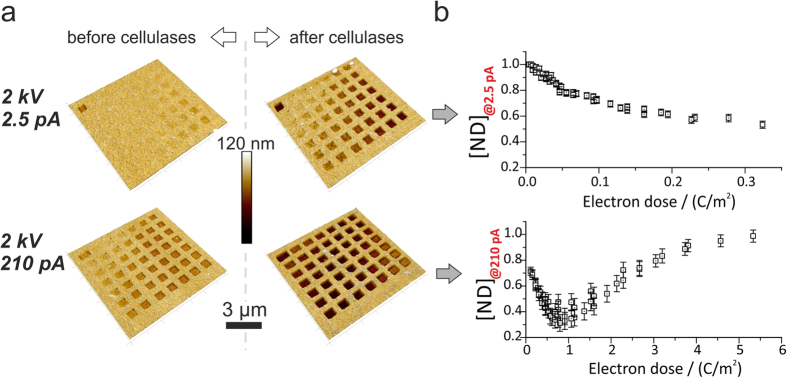
(**a**) AFM height images of test patterns before (left) and after enzyme exposure (right) for low and high beam currents of 2.5 pA (top) and 210 pA (bottom), respectively (2 keV primary energy). The different fields correspond to different electron doses via DT and frame number variations (see supplement 2). (**b**) Summary of the degraded volume fraction after enzyme exposure in dependency on the applied dose (see supplement 2). Note, this relative representation has been chosen for further correlation with the theoretical model.

Based on the above mentioned observations, we transferred the FEBIC process to a dual-beam instrument (NOVA 200, FEI, The Netherlands) which provides a high-performance patterning engine for precise control of process parameters in order to determine ideal conditions for full conversion. Variables of interest are electron energy or beam voltage (**U**_**Beam**_), beam current (**I**_**Beam**_), pixel dwell-time (**DT**) and the pixel point-pitch (**PP**) between two consecutive patterning points. To allow comparable calculation of the applied electron doses, we kept the PP equal to 50% beam overlap in dependence on the beam diameter (see supplement 2 and 3). For each set of U_Beam_ and I_Beam_ (12 combinations in total) a 7 × 7 matrix of 1 × 1 μm^2^ fields has been structured on 100 nm thick TMSC films on SiO_2_ / Si (5 nm / bulk) substrates with a systematic variation of DTs and frame-numbers (exact layout can be found in supplement 2). Subsequently, the structured films were immediately subjected to AFM imaging in ambient conditions for reference measurements (a graphical work-flow diagram concerning the experimental strategy can be found in supplement 2). Afterwards, the samples were exposed to a cellulase cocktail (produced by Hypocrea jeronica sp.) for 24 hours at 30 °C. (see experimental section for details). Finally, AFM was used again for detailed morphological characterization to quantify the bio-degraded material. To exclude the possibility of water swollen cellulose, respective films were carefully dried before post-incubation AFM measurements. Please note that Rehfeldt and Tanaka^51^ demonstrated in dynamic experiments that film height is conserved before and after waters swelling. Figure 1a shows AFM height images of a parameter matrix before (left) and after enzyme incubation (right) structured at 2 keV beam energy with low (2.5 pA; Fig. 1a top) and high beam currents (210 pA; Fig. 1a bottom). The first remarkable detail is a dose dependent volume loss directly after patterning (left images). This is in agreement with previous findings by Kontturi and Lankinen^47^ which reported a volume loss of up to 50% due to the loss of larger TMS groups upon regeneration to cellulose. The second detail is the clear volume loss after enzymatic incubation (right images) which has been quantified in a relative fashion (Fig. 1b). Here, each enzyme degraded pattern is normalized to its former height, thus specifying the amount of non-degradable (**ND**) material. Figure 1b shows the relative volume loss in dependency on the applied electron dose calculated from the constant process parameters I_Beam_, PP and the variable DT. As evident, there is a clear minimum for the high-current sample (Fig. 1b; bottom) slightly below 1 C/m^2^ electron dose followed by an increase, which indicates that higher doses might over-convert the TMSC into ND materials (discussed in detail later). For the lower beam current (Fig. 1b; top) we see no minimum but a steadily decreasing branch which, however, simply stems from too low doses (<1 C/m^2^) presumably required for ideal conversion (see 210 pA experiments). To investigate whether this behavior is generally valid, we expanded the experiments (*structuring* → *AFM* → *incubation* → *AFM*) to all combinations of U_Beam_, I_Beam_, PPs and DTs.

**Figure 2:**
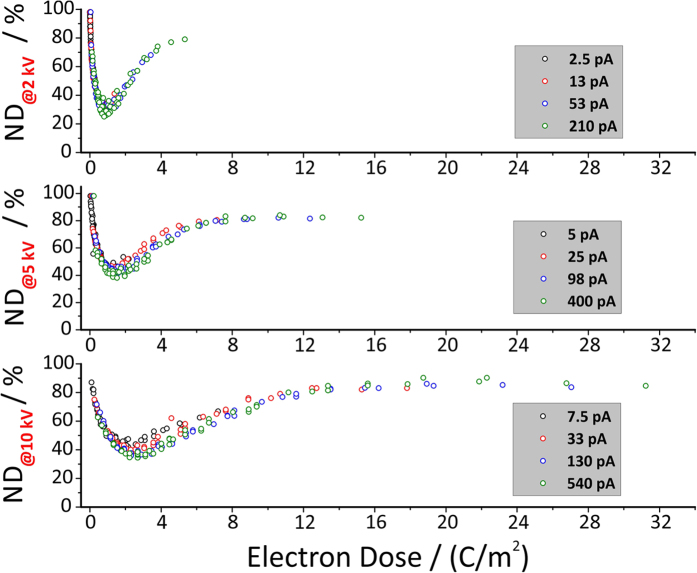
Absolute height loss after enzymatic incubation for 24 hours at 30 °C specifying the evolution of non-degradable material (ND). As evident, for a given primary electron energy U_Beam_ the behavior is predominantly dose dependent and barely affected by different beam currents and / or patterning parameters. The residual height, even at optimal doses, represents a characteristic feature of the presented approach as a consequence of simultaneously concurrent chemical reactions by means of TMSC → cellulose (wanted) and cellulose → non-degradable material (unwanted) as described in the main text.

**Figure 3:**
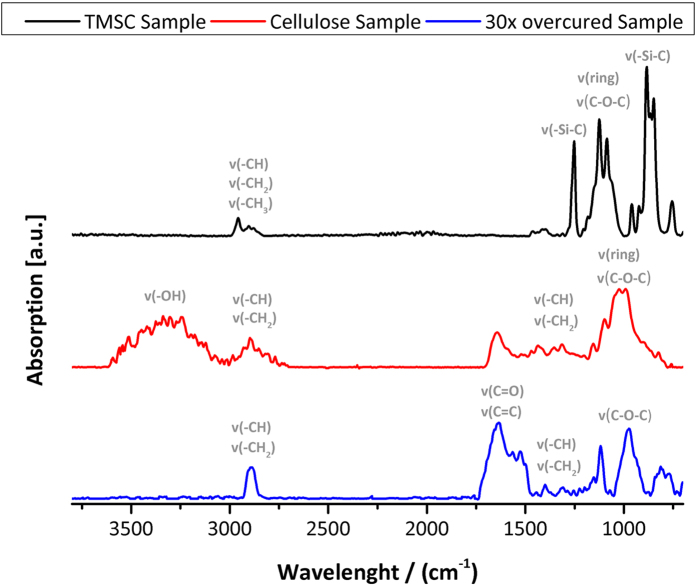
ATR-IR spectra of TMSC and focused electron beam processed cellulose films. The TMSC spectra after spin coating is shown on top (black) with clearly identifiable -Si-C bands at 849–883 cm^−1^ and 1252 cm^−1^. After ideal conversion (center, red), no –Si-C bands are visible and clear cellulose bands at 3303 cm^−1^ (-OH) and 990–1032 cm^−1^ (-C-O-C-; -C-O-) are found. Dedicated experiments via 30 fold over-conversion (bottom; blue) proves the significant beam damage to the former generated cellulose structure. Here as proposed, no cellulose typical -O-H bands are visible. Spectra were obtained via a total of 1024 scan on a 100 x 100 x 0.6 μm^3^ patch on gold covered SiO_2_ substrates. The extremely low amount of material accounts for the low signal to noise ratio.

Figure 2 summarizes the results and shows the absolute height losses after enzyme exposure in dependence on the applied doses. Please note, as different beam energies imply different penetration depths of the electrons the graphs have been separated accordingly. As evident from the results, each minimum mainly depends on the applied dose (at same primary energies) and is widely independent on the used beam currents and patterning parameters. It is known from literature that the applied enzyme cocktail is incapable to degrade TMSC with a substitution grade larger than 0.5^26^. Therefore, we can draw 2 conclusions: ***1)*** the electron beam indeed converts TMSC into cellulose with ***2)*** an dose dependent conversion efficiency. Although the degradation effect itself is a very strong indication for a successful *TMSC *→ *cellulose* conversion^26^,^52^, further evidence is required that the intermediate product is pure cellulose. Therefore, we conducted FT-IR spectroscopy investigations on 200 × 200 μm^2^ structured cellulose patches which have been structured at optimal doses (10 kV, 130 pA, 800 ns DT). Figure 3 shows spectra of TMSC (top, black), ideally converted FEBIC cellulose (center, red) and over-cured films (bottom, blue). The latter were exposed to a 30 fold electron dose and show no resemblance with the optimal cured patches (red). As expected no bands for the -O-H vibration are found in the over cured films while an increase in C=C vibrational bands is observed. This is in well agreement with the hypothesized beam damage of the formerly regenerated cellulose. The optimal dose patterns show a well resolved cellulose spectrum with the typical -O-H and -C-O-C bands^53^,^54^. More strikingly, however, is the absence of any TMSC residues which finally confirms full conversion into cellulose via focused electron beams in agreement with the enzymatic degradation experiments. Please note, FEBIC processes are only ideal for patterning fields up to a few tens of microns. Hence, the investigated regions are first small and second very thin which explains the low signal-to-noise ratio in the spectra.

**Figure 4:**
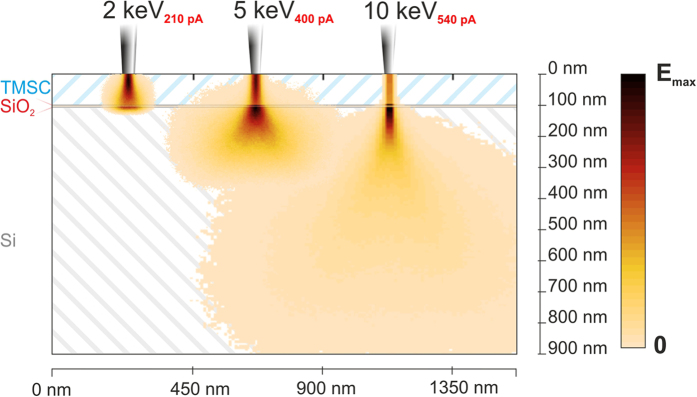
Monte Carlo simulation^59^ of deployed energy for 2, 5, and 10 keV electrons in the x-z plane. The color of each pixel is the cumulative sum over the y-coordinate of deployed energy within this pixel. For 2 keV approximately 72.5% of the primary energy are deployed within the TMSC layer; For 5 keV and 10 keV primary energy this factor is reduced to 33.7% and 12.8%, respectively, which is in good agreement with the scaling factor of required doses for ideal conversion Fig. 2. Please note, backscattered electrons are not included in this visualization for more clarity.

In summary, direct chemical measurement and indirect enzymatic degradation show that optimally regenerated material is indeed cellulose without impurities from TMSC. Now, we can reconsider Fig. 2 and classify the observed behavior into 3 conversion regimes: ***1)*** electron-limited-regime (**ELR**) for low doses which lead to incompletely converted TMSC; ***2)*** optimum-regime (**OR**) for ideal conversion; and ***3)*** electron-excess-regime (**EER**) for high electron doses. First, we discuss the EER regime which converts the TMSC in non-degradable ND material (towards zero volume loss in Fig. 2). We attribute this over-conversion to classical electron beam damage of polymers^55^,^56^,^57^ which is a well-known problem in electron-microscopy. Due to the strong evidences that ideal doses convert TMSC into cellulose (OR), it logically follows, that for very low doses an incomplete conversion takes place. This is consistent with the reduced height loss at low doses as the applied enzymes are incapable to degrade TMSC with a degree of substitution (DS) higher than 0.5^26^. Concerning the conversion itself, we refer to fundamental processes during FEBID processes, where low-energy electrons cause radiolysis of precursor molecules to a deposited and intended material^41^,^42^,^43^. TMSC usually requires acidic components to resubstitute the TMS moieties by hydrogen^46^,^58^. For FEBIC processes we hypothesize, that ionization effects and thus secondary electron generation provide a sufficient amount of H^+^ for re-substitution. Here, further investigations are clearly needed to identify the responsible effects and origin of the required proton, which, however, is not in conflict with the present work as we have provided the evidence that the intermediate product is cellulose. The final detail to be explained concerns the increasing ideal electron dose for increasing beam energies as evident in Fig. 2. It is well known that higher primary electron energies entail higher penetration depths and feature larger so called interaction volumes. As a consequence, the content of “available” electrons within the TMSC film is decreasing for higher electron energies. Fig. 4 shows a Monte Carlo simulation of the mean energy loss in each pixel weighted with the electron energy at entry (Casino 2.48, Universite de Sherbrooke, Canada)^59^. The graph illustrates the situation for a 100 nm thick TMSC film on SiO_2_ / Si (5 nm / bulk) substrate in a cross-sectional view. While for 2 keV electrons most electrons of a single pulse remain in the TMSC layer, a majority of 10 keV electrons are found in the substrate. More detailed calculations reveal an energy loss in TMSC of 72.5% for 2 keV while 33.7% and 12.8% were found for 5 keV and 10 keV electrons, respectively. This roughly correlates with the scaling factor for increasing optimal doses in Fig. 2. Please note, for an exact determination, the dissociation cross-section of TMSC would be needed which is not available to date. Please note, a definite number concerning the ideal primary electron energy cannot be given as this criteria depends on the TMSC film thickness. To provide values as a starting point for successful reproduction, supplement 6 gives a table of minimum electron energies in dependency on the initial TMSC film thickness.

### Conversion Process

In conclusion, the systematic characterization suggests a two phase process: ***1)*** conversion TMSC → cellulose; and ***2)*** cellulose → non-degradable carbon rich material which might be written in a two-step chemical formula:




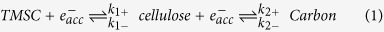




As a matter of fact, reaction constants k_1−_ and k_2−_ may be neglected as these are not likely to happen. Furthermore, electrons are constantly supplied by the electron beam and thus not diminished during individual beam pulses (DT). Such reactions may be described by pseudo-first order chemical reactions^60^. Using this formalism (full derivation can be found in supplement 4) we can deduce physical relevant fitting functions to obtain valuable parameters:




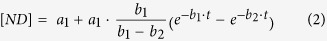




**Figure 5:**
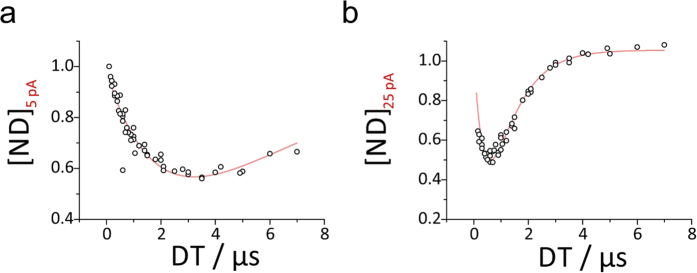
Experimental data of non-degraded material for 5 keV electrons at 5 pA (**a**) and 25 pA (**b**) beam current. The fit is shown in red and describes experimental data considerable well. Tabulated values of fit parameters and similar curves for each pair of U_Beam_ and I_Beam_ may be found in supplementary Table S3–S4 and supplementary Figure S4–S7.

**Figure 6:**
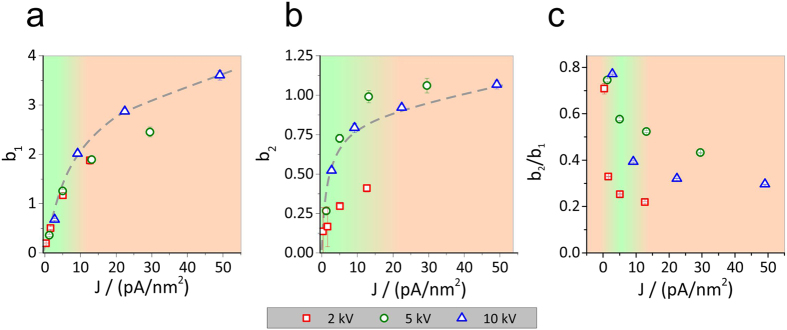
Fitting data for parameters b_1_, b_2_ and the ratio of b_2_ / b_1_ plotted against the current density. Data for b_1_ and b_2_ may be related to the reaction rate and shows that b_1_ is significantly higher than b_2_ which is extremely important for successful regeneration to cellulose. Moreover, exceeding current densities of 10 to15 pA/nm^2^ shows saturation tendencies for b_1_ and b_2_, respectively, which is expectable concerning the limited number of reaction sites.

Here, a_1_, b_1_ and b_2_ are fitting parameters and correspond to the normalized concentration of TMSC and to products of rate constants k_1+_ and k_2+_ with the concentration of electrons, respectively. The intention to shape this equation this way is the possibility to fit experimental curves in Fig. 4. Formulation of equation (2) bases on an assumed similar concentration of conversion relevant electrons for both reaction phases. This assumption is justified as the increasing material density is compensated by the volume loss during conversion as shown by Kontturi, Lankinen and Ehmann *et al.*^47^. Briefly, they used X-ray reflectivity to determine the increase of density from 0.99 g/cm^3^ to 1.51 g/cm^3^ and the corresponding decrease of film thickness by 50% during the regeneration of TMSC with hydrochloric acid vapors. The same data was used in Monte Carlo simulations as depicted in Fig. 4 and showed that energy loss within TMSC and denser but thinner cellulose films differ only by a few percent. *Hence, b*_*1*_ *and b*_*2*_ *are directly related to k*_*1*_ *and k*_*2*_. Applying now equation (2) to experimental data as shown in Fig. 1b, we can fit the curves to achieve the corresponding parameters and thus test the proposed two-phase process (equation (1)) on its validity. Figure 5 representatively shows such fits for 5 keV electrons for low (5 pA, (a)) and higher beam currents (25 pA, (b)). As evident, the proposed function (equation (2)) describes experimental data exceptionally well over all three regimes for 5 keV but also holds for all other pairs of U_Beam_ and I_Beam_ as shown in detail in supplement 4 (supplementary Figure S3–S6). Concluding, these results show the validity of the presented mathematical model of a two phase process. While we have already shown before that the intermediate and desired product is cellulose, beam damage also present from the beginning renders a fraction of the exposed materials non-degradable. The assumption of a carbon-rich residue at high doses is feasible as similar processes in FEBID and dedicated studies have been shown in literature^57^,^61^. The fitting model provides valuable information on the *rate-constants* and *reaction-speeds* (full summary can be found in supplement 4). Particularly interesting are the parameters b_1_ and b_2_ which are equivalent to reaction-rate constants for the first (TMSC → cellulose) and the second process (cellulose → ND carbon-rich material). Figure 6 shows both parameters against the current density for all beam energies used. Please note, a plot against the beam current may lead to misleading results, as each current has different beam profiles. First thing to notice is that both parameters (b_1_ and b_2_ in (a) and (b), respectively) saturate for higher current densities. This is expectable due to a limited number of relevant bonds in the TMSC / cellulose films which at some point is exceeded by the number of introduced electrons. Thus, fastest rates are achieved at current densities close to the transition point while higher doses provide much more electrons than required and initiate EER conditions with strong formation of ND carbon material. For the TMSC → cellulose reaction (b_1_ and thus k_1_), this threshold lies around 15 pA/nm^2^ while a value of about 10 pA/nm^2^ is found for the proposed cellulose → ND carbon reaction (b_2_ and k_2_).

Several more and conclusive details can be extracted from these graphs. First, it is evident that absolute values of b_1_ are much higher than for b_2_ (~factor 3). This not only means that TMSC → cellulose reactions are faster but also explains the asymmetric behavior in Fig. 2. Second detail is that, although b_1_ reaches larger values, the increase of b_2_ is significantly faster. This provides evidence for radiation damage as soon as cellulose fragments are available. This nicely explains why it was not possible to degrade 100% of the structured fields as can be seen in Fig. 2. In this respect it is of advantage to consider the ratio b_2_/b_1_ which reflects the balance between both reactions. Figure 6c shows this ratio in dependency on the used beam current densities for different primary energies. As evident, the lowest values are found for lowest primary beam energies, which means, that the first and intended reaction (TMSC → cellulose) is dominating. This is in consistency with the observation that 2 keV structures resulted in highest volume losses (see Fig. 2). This is also the first indication that lower energies seem to be more appropriate for a fast and more complete conversion into cellulose. Please note, the exact value of the primary energy ultimately depends on the TMSC layer thickness which should be in the same range as the vertical interaction volume dimension (see supplement 6). Thus thicker films should be structured with tuned primary beam energy which can be evaluated by the use of Monte Carlo simulations (Casino 2.48; Fig. 4) 59. Another detail in Fig. 6c is the observation that all b_2_/b_1_ ratios decrease with higher beam currents. This means that very low currents entail higher contents of unwanted ND carbon generation and therefore should be avoided. Although minor, this effect can nicely be seen in Fig. 2 for 10 keV structures where lowest currents lead to less degradable cellulose. Hence, in summary with data from Fig. 6a,b where saturation is found after approximately 10–15 pA/nm^2^, we can state from a chemical point of view, that lower beam energies and intermediate beam currents are beneficial concerning the ideal cellulose conversion. With this elaborate and comprehensive analysis of reaction kinetics, reaction yield and evaluation of the corresponding chemistry, we proceeded by evaluating the highest attainable resolution.

### Downscaling

**Figure 7:**
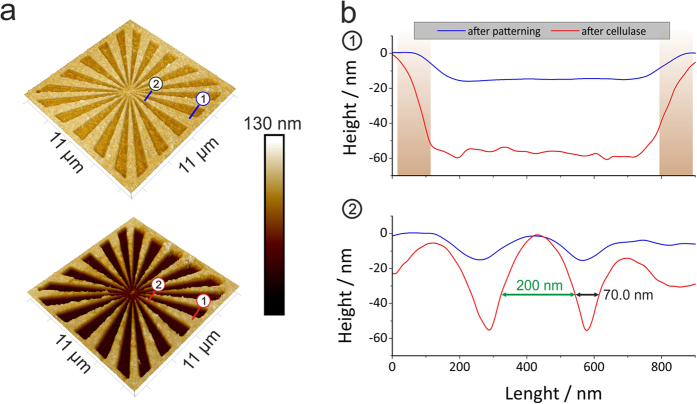
A FEBIC structured TMSC film visualized by AFM (**a**) imaging before (top) and after enzymatic treatment (bottom). In (**b**) section profiles near the center (2) show the minimal line width of 70 nm and a minimum distance of 200 nm. Near the edge (1) BSE effects lead to edge broadening effects as evident by the comparison of patterned and enzyme treated section lines (see brown shading).

For this purpose, we designed different patterns via black / white bitmaps which were further converted into interlacing stream files for direct use with the dual beam patterning engine^62^,^63^,^64^. To test the resolution capabilities, pattern geometries as depicted in Fig. 7 have been chosen together with process parameters of 2 keV primary electron energy, 53 pA beam current and DTs of 1500 ns to achieve ideal doses at optimum b_2_/b_1_ conditions. The decreasing line and space widths allow accurate analyses of the minimum distance of two un-structured areas and line-widths in between. Figure 7a shows the structured areas after patterning (top) and after enzymatic degradation (bottom). Multiple measurements on several samples revealed full-width-at-half-maximum (FWHM) line- and space widths of below 70 nm and 200 nm, respectively, as representatively shown by cross-sectional profiles in Fig. 7b (taken from indicated regions in Fig. 7a). These values can be rationalized by taking the back scattered electrons (**BSE**) into account as well. For structuring points at the pattern edge, this electron species leads to an intrinsic broadening effect as indicated by the brown shading in Fig. 7b (1). The exact broadening width is determined by the layer chemistry (TMSC) and the applied primary energy as studied in detail by Schmied *et al.* and Arnold *et al.*^65^,^66^ for FEBID nano-structures. Hence, BSE proximity effects ultimately limit the achievable resolution for unstructured areas (Fig. 7b (1)). In contrast, fully converted regions can be made much smaller as BSE effects are of minor relevance for the patterned regions as confirmed via the cross-sectional profile in Fig. 7b (2). This immediately implies that lowest primary electron energies have to be used to minimize the BSE related broadening effect for highest lateral resolution. However, the thickness of the TMSC layer has to be taken into account in such a way that the according interaction volume should entirely penetrate the precursor layer as shown in Fig. 3 for the 2 keV situation (see supplement 6). Lower energies would lead to unaffected TMSC regions at the bottom. In contrast, higher energies lead to electron-substrate interactions which entail substrate related BSE effects which further decrease the achievable lateral resolution (detailed analyses is found in supplement 5). By that, it can be stated that highest lateral resolution is achieved when the vertical dimension of the interaction volume fits to the TMSC layer thickness. This simply requires an initial simulation to find ideal primary electron energies with respect to the TMSC layer of interest. Concerning the ultimate FEBIC resolution it is expectable that very thin films might allow feature sizes below 100 nm for both line and space. The chemical limit might be given by the length of a typical cellulose chain which typically consist of hundreds of unit moieties leading to edge roughening (intrinsic limit).

## Conclusions

In this study, we introduced focused electron beam induced conversion (FEBIC) as a feasible, mask-less, direct-write method to convert a cellulose precursor (TMSC) into cellulose with lateral resolution in the sub−100 nm regime. During conversion, we identified three regimes denoted as *electron-limited*, *optimal-* and *electron-excess*-*regime* (ELR, OR, EER). While ELR is characterized by incomplete TMSC → cellulose conversion, EER conditions lead to non-degradable (ND), non-cellulosic material due to overexposure. In the OR regime maximum regeneration of TMSC into cellulose is established as confirmed by direct and indirect experiments using FT-IR and enzymatic degradation, respectively. An elaborate modeling of the corresponding reaction mechanisms using pseudo-first order kinetics revealed a two-step conversion by means of *TMSC* → *cellulose* and *cellulose* → *ND materials*. The correlation with experimental data not only revealed excellent agreement but also allowed a deeper insight in reaction dynamics. It was found that lowest possible energies and intermediate beam currents are best suited for fastest conversion rates and highest volumetric conversion degree. Although in well agreement with experimental data, the exact reaction mechanisms are yet not understood in detail. In similarity to electron induced radical formation on water molecules^49^, FEBIC is likely to cause multiple reaction pathways including silyl radicals, trimethylsilanol and hexamethyldisiloxane. Therefore, further studies are required to unravel the corresponding reaction products for a comprehensive process understanding. Finally, downscaling experiments revealed that converted areas below 100 nm can be achieved for ideal settings. A more detailed look further strengthened the demand for lowest possible primary electron energies to prevent any proximity effects from the underlying substrate. By that this study introduced a new approach for the defined structuring of cellulose with sub−100 nm resolution for the combination with electronic devices, microfluidic arrays, small scale bio-sensors or diagnostic tools. Finally, it should be mentioned that the structuring of chitin based films is now feasible which show a slightly different but highly interesting chemistry^67^ for fundamental research.

## Methods

### Materials

All used materials and chemicals have been purchased in highest available purity unless otherwise stated. Pre-cut silicon wafers (10 × 10 mm^2^) with 5 nm SiO_2_ were kindly provided by AMS AG (Unterpremstätten, Austria). Glass vials (4 mL, Ø 15 mm, Rotilabo), microscopy slides, 2-propanol, ethanol (analytical grade, not denatured) and xylol were purchased from Carl Roth (Karlsruhe, Germany). Trimethylsilyl-cellulose (TMSC, DP = 2.8) was purchased from Thüringisches Institut für Textil- und Kunstoff-Forschung (TITK e.V, Germany).

### Preparation of Trimethylsilyl-cellulose films

Cellulose films were prepared according to protocols from literature^7^,^58^. Briefly, 20 mg·ml^−1^ of TMSC were dissolved in xylol and transferred to a sonification bath (Transsonic T560, Elma Schmidbauer GmbH, Germany) and treated until no residual particles were observable (typically 15 min). The resulting solution was drawn into a syringe and filtered through a nitro-cellulose filter with a nominal pore size of 5 μm into a new capped glass vial. In a next step, silicon wafers (AMS AG, Unterpremstätten, Austria) were carefully removed in a flow box to prevent contamination with dust and transferred to the spin-coater (Laurell ws-650-S7-6NPP/LITE, Laurell Technologies Corporation, NW, USA). Approximately one hundred to two hundred μl of solution were pulled up into a glass pipette and transferred onto the silicon specimen, followed by immediate spin-coating. Parameters were: An acceleration period of 4 seconds to 3600 rpm, followed by constant spinning for further 25 seconds to ensure complete evaporation of the solvent. Specimens were removed from the coater and stored until further use in Parafilm sealed petri-dishes.

### Focused electron induced regeneration

TMSC thin film specimens were positioned on a conventional SEM holder (Ø 10 mm) by double sided adhesive carbon tape. A FIB Nova 200 microscope (FEI Company, The Netherlands) was used for the patterning of the TMSC thin films. Optimal parameter range was analyzed according Table S1 and Figure S2 as specified also in the results section. Here U_Beam_, I_Beam_, DT and PP were varied to find a set of optimal patterning parameters. Once optimal parameters were available, the patterning was performed as follows: For each structure, conventional drawing tools (CorelDraw X6, Corel Corporation, Canada) were used to design a black/white bitmap image with the corresponding non-patterend/patterned points, respectively. The image was then processed by the recently introduced SIL engine to gain a corresponding stream file^62^. Briefly, this engine was specially designed to minimize the thermal stress during FIB processing which is of essential relevance for low melting materials such as (bio-) polymers. Patterns were then structured in the specimens by the FIB patterning engine. Please note that the structuring was applied in “blind” mode as each electron would lead to regeneration effects. In this context the e-beam was blanked immediately before and after patterning within 20 ns. After patterning, specimens were removed from the vacuum chamber and stored in Parafilm capped petri-dishes for further characterization or further processing.

### Enzymatic hydrolysis

Complete cellulase system of *Hypocrea jeronica* mutant SVG 17 was prepared according to protocol from literature^68^. All hydrolysis experiments were performed using 2 ml of 50 mM sodium citrate buffer (pH 5.0) and 300 μl of the cellulose supernatant (0.1 FPU/ml). Hydrolysis was performed at elevated temperatures (30 °C) and for 24 hours to ensure complete conversion of degradable material. Preliminary experiments confirmed complete degradation after a maximum time of 19 hours (the major part was already degraded after about 2 hours). Afterwards, specimen was carefully rinsed with deionized water for 5 minutes, followed by CO_2_ spray drying.

### Attenuated total reflection infrared spectroscopy (ATR-IR)

For ATR-IR experiments, silicon wafer specimens were preliminary covered with a 10 nm layer of chromium followed by 100 nm of gold. TMSC films were then prepared and structured according to the procedures above. For ATR-IR experiments an area of approximately 100 × 100 μm^2^ was fully regenerated by the electron beam at the optimal parameters (U = 2 kV; I = 53 pA; DT = 1200 ns; P = 1; PP_@50%overlap_ = 10.4 nm). The experiments were performed with an ALPHA FT-IR spectrometer (BRUKER; MA, USA). For the measurement an attenuated total reflection (ATR) attachment was used with 48 scans at a resolution of 4 cm^−1^ and a scan range between 4000 and 400 cm^−1^. The data were analyzed with OPUS 4.0 software.

### Atomic force microscopy

AFM investigations were carried out using a FastScan Bio AFM microscope (Bruker AXS, CA, USA) operated by a Nanoscope V controller. For all investigations FastScan C cantilevers (Bruker AXS, Santa Barbara, CA / USA) with nominal spring constants of 0.8 N/m and a tip radius of 5 nm were used. Experiments were conducted under ambient conditions at an air conditioned temperature of 20 °C. Films were analyzed in negative or positive structured manner, that is with still present TMSC layer or without, respectively. In order to produce the positive structures, films were immersed in xylol for 2 minutes prior to AFM measurement in order to remove the TMSC. For negative structured films carful scratching with ultra-sharp tweezers allowed a reference to the underlying silicon for height measurement. Setpoints, scan rates and controlling parameters were chosen carefully to ensure lowest possible energy dissipation to the sample and to exclude tip driven artifacts. Data analysis of images was performed using Nanoscope Analysis 1.50 (Build R2.103555, Bruker AXS, CA, USA) and Gwyddion 2.38 (Released 2014-09-18, http://gwyddion.net/). All images were plane fitted at 1^st^ order unless otherwise stated.

## Additional Information

**How to cite this article**: Ganner, T. *et al.* Direct-Write Fabrication of Cellulose Nano-Structures via Focused Electron Beam Induced Nanosynthesis. *Sci. Rep.* **6**, 32451; doi: 10.1038/srep32451 (2016).

## Supplementary Material

Supplementary Information
