# Supplementary material for: Direct-Write Fabrication of Cellulose Nano-Structures via Focused Electron Beam Induced Nanosynthesis
Source: Sci Rep. 2016 Sep 2;6:32451. doi: 10.1038/srep32451 (PMC5009462; doi:10.1038/srep32451)
Supplement: Supplementary Information [file srep32451-s1.pdf]

Supplementary Information

Direct-Write Fabrication of Cellulose Nano-Structures via Focused Electron Beam Induced Nanosynthesis.

Thomas Ganner[1], *Jürgen Sattelkow*[1], *Bernhard Rumpf*[1], *Manuel Eibinger*[4], *David Reishofer*[3], *Robert Winkler*[2], *Bernd Nidetzky*[4,5], *Stefan Spirk*[3,†], *Harald Plank*[1,2*]

[1] Institute for Electron Microscopy and Nanoanalysis, Graz University of Technology, Steyrergasse 17, A-8010 Graz, Austria

[2] Graz Centre for Electron Microscopy, Steyrergasse 17, A-8010 Graz, Austria

[3] Institute for Chemistry and Technology of Materials, Graz University of Technology, Stremayrgasse 9, 8010 Graz, Austria

[4] Institute of Biotechnology and Biochemical Engineering, Graz University of Technology, Petersgasse 12, A-8010 Graz, Austria

[5] Austrian Centre of Industrial Biotechnology, Petersgasse 14, A-8010 Graz, Austria

* Corresponding author: harald.plank@felmi-zfe.at

† Co-corresponding author: stefan.spirk@tugraz.at

1 Preliminary experiments (H_2O dependence)

Geier and coworkers [1] demonstrated that the electrolysis of H_2O is a key requirement for purification of carbon rich metal precipitates initially fabricated via Focused Electron Beam Induced Depostion (**FEBID**). We therefore conducted preliminary experiments using QUANTA 200 ESEM (FEI, The Netherlands) in high-vacuum (2.10^{-5} mbar chamber pressure) and low-vacuum mode with 10 Pa H_2O partial pressure at room temperature. Prior to this exposure, we fabricated 100 nm thick TMSC films on SiO_2 / Si (5 nm / bulk) substrates via spin-casting and immediately transferred them to the electron microscope. Results obtained by light microscopy are shown in Figure S 1. The left parts gives the patterned square regions after e-beam exposure while the right parts show same areas are 24h incubation to the highly specific enzyme cocktail *Hypochrea jeronica* (parental strain: RUT-C30). As evident, low-vacuum H_2O conditions led to massive proximity effects which prevent high-resolution structuring as a consequence of the well-known skirt scattering effects in ESEMs. For high-vacuum conditions, however, no such proximity effects have been observed while the enzyme cocktail entirely removed the patterned areas. Based on the high specificity of the applied enzyme cocktail to pure cellulose and the fact that surrounding, pristine TMSC areas were entirely unaffected, we hypothesized that e-beam induced conversion is a pathway for defined high-resolution cellulose structuring. Based on these preliminary experiments, we investigated the process in more detail which represents the main part of this manuscript.

[1] Geier, B.; Gspan, C.; Winkler, R.; Schmied, R.; Fowlkes, J. D.; Fitzek, H.; Rauch, S.; Rattenberger, J.; Rack, P. D.; Plank, H. Rapid and Highly Compact Purification for Focused Electron Beam Induced Deposits: A Low Temperature Approach Using Electron Stimulated H 2 O Reactions. *J. Phys. Chem C* **2014**, *118*, 14009–14016.

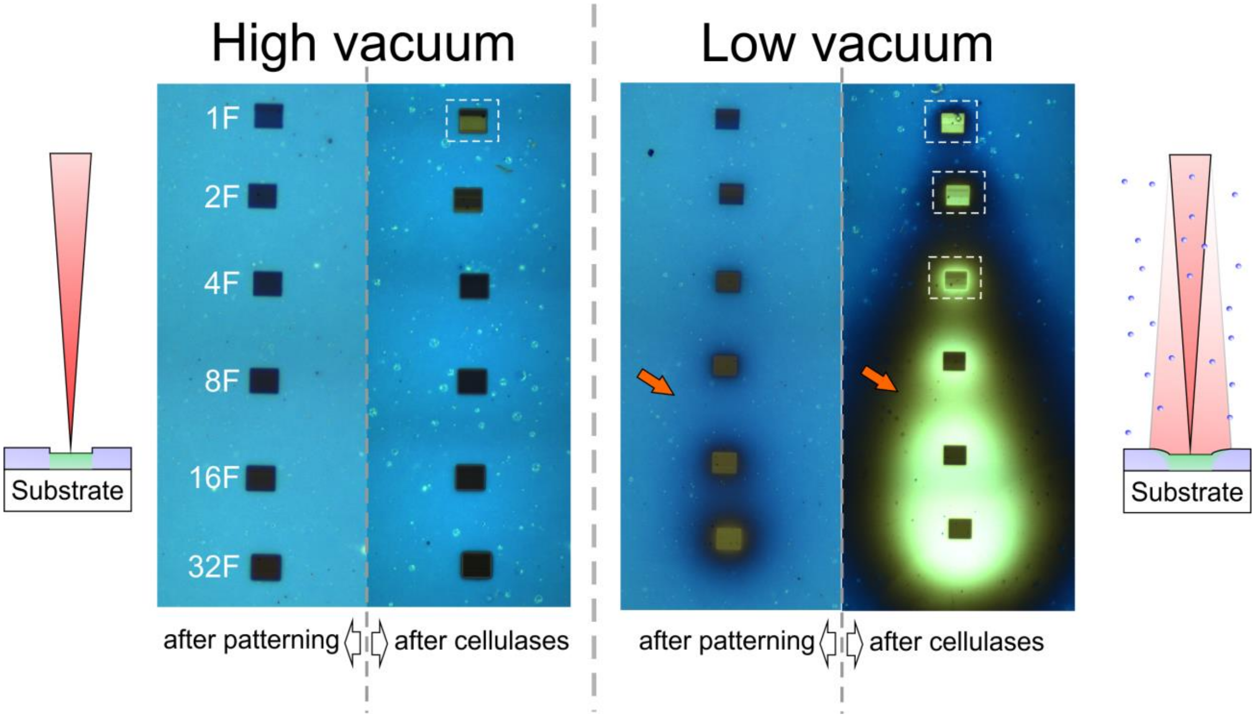

Figure S 1: Preliminary conversion experiments in an ESEM. Experiments were conducted in HV (left part) or LV mode with 10 Pa H_2O partial pressure (right part). Acceleration voltage was 10 kV with a measured beam current of 778 pA, a frame size of 5 x 5 µm^2 at 3584 x 3094 pixels (which corresponds to a point pitch of approximately 1.6 nm) and a dwell time of 100 ns per pixel. In HV mode only the 1 frame pattern (1F, white dotted rectangle) shows a different layer height after cellulase treatment while in LV mode a change is seen up to 4 frames (4F, white dotted rectangles). The reason is simply, that skirt effects (schematic right site) lead to significant spreading of the particle beam which further leads also to degradation effects (see orange arrows).

2 FEBIC Patterning-parameters and -layout

In the following the applied structuring layout is specified in detail. For all experiments 100 nm TMSC films have been used which were spin-cast on SiO_2 / Si (5 nm / bulk) substrates. For each pair of U_{Beam} and I_{Beam} (12 combinations in total, see Table S1), a systematic variation of dwell-time (DT) and frame-numbers (multiplicator) have been applied (see Figure S2). All pads were 1x1 μm^2 in size with 1 µm distance in between. The point-pitch (PP) was defined by a 50% beam overlap accounting for the fact that beam profiles change with voltage and current. This simplifies the dose calculation for further modeling.

Table S 1: Used electron acceleration voltages (U_{Beam}) and beam currents (I_{Beam}) in the parameter sweep experiments.

	**U = 2 kV**	**U = 5 kV**	**U = 10 kV**
**I_1 (pA)**	210	400	540
**I_2 (pA)**	53	98	130
**I_3 (pA)**	13	25	33
**I_4 (pA)**	2.5	5	7.5

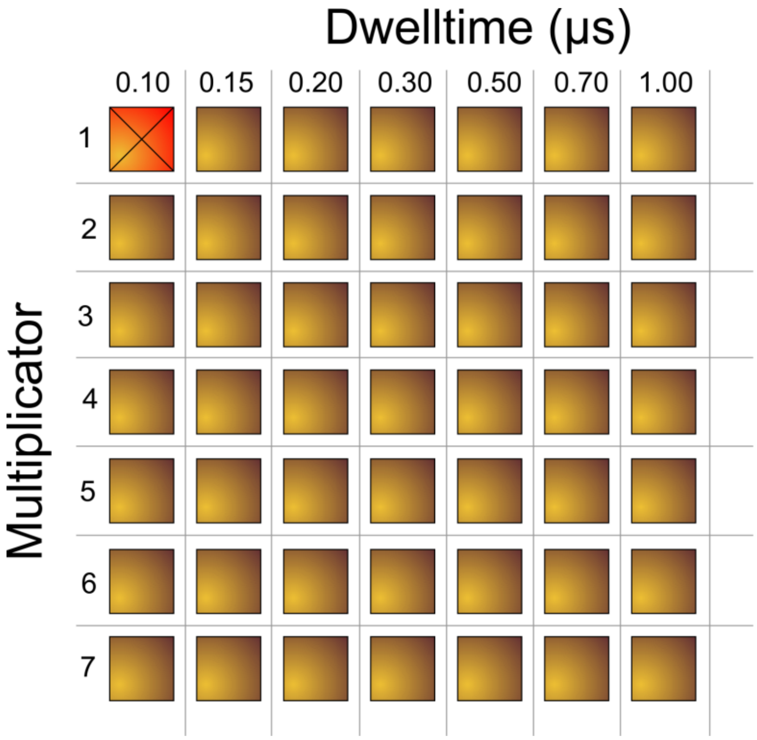

Figure S 2: Dwell-times (DT) and multiplication values (multiple frames) used for each acceleration voltage and beam current as specified in Table S1. The obtained pattern therefore is representative for different introduced electron doses (see equation 1 and 2). Note, preliminary investigations already defined roughly the borders between DTs of 0.1-7 µs. The red rectangle is patterned at DT 4 µs and acts as synchronization and positioning pad.

To clarify the experimental strategy used throughout this study, a work-flow chart can be found in Figure S3. As evident, the procedure starts with the (1) preparation of the TMSC films (as specified in the methods section of the main manuscript) followed by (2) the e-beam structuring (FEBIC) and (3) AFM height analysis after patterning (as prepared), then subjected to (4) the enzyme incubation with cellulases which removes transferred cellulose (conditions are again specified in the methods section of the main manuscript) and (5) finally analyzed via AFM height analysis after enzyme incubation. By using the calculation $(h_{be}-h_{ae})/h_{be}$ we are able to specify the non-degradable material in a relative fashion. The process is finished with a fitting routine as depicted in **supplement 4**.

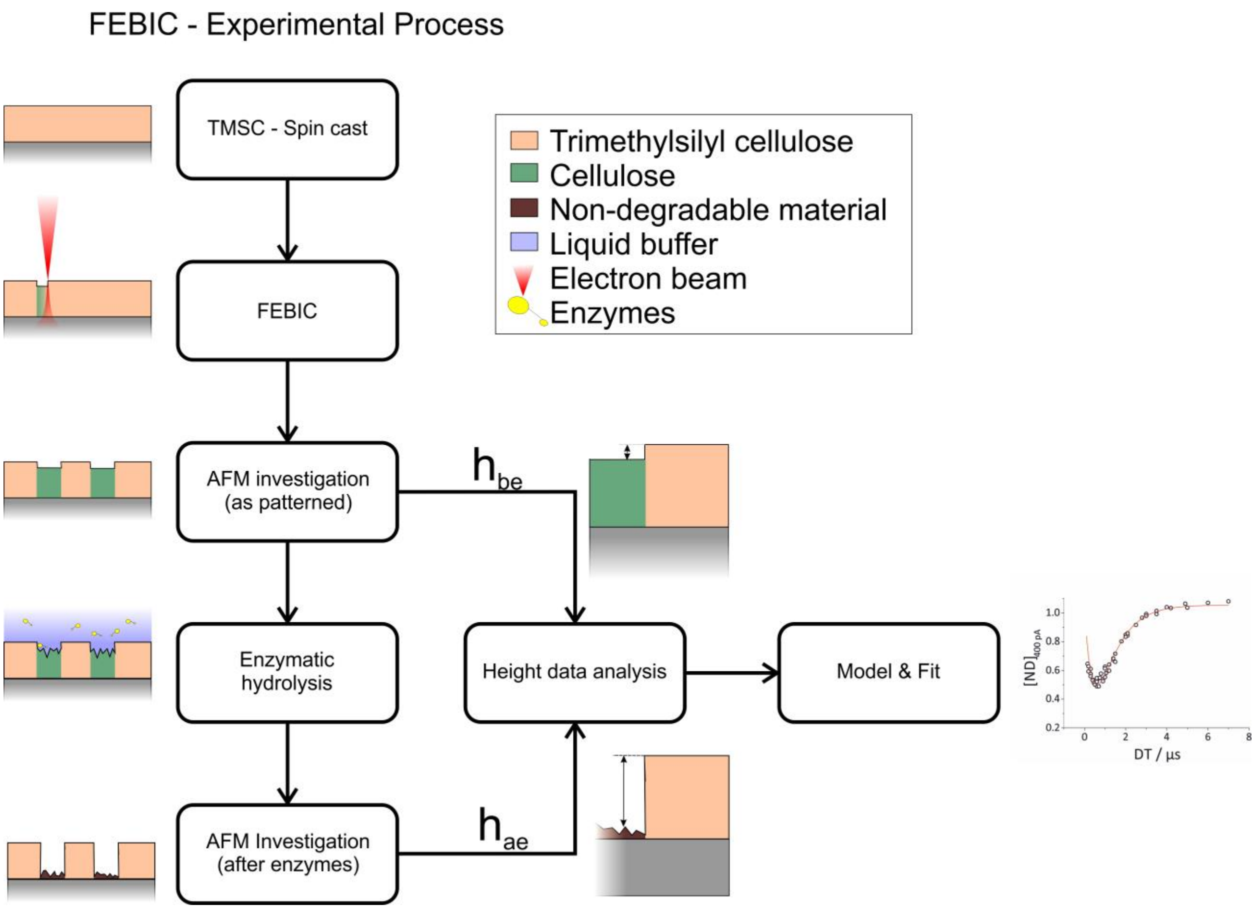

Figure S 3: Schematic representation of the experimental work flow.

3 Calculation of the electron dose

The applied electron dose (D) is calculated according to equation (1) and (2). Starting from the universal equation 1 the dose D can be re-written as shown in equation (2). In this special case we can substitute the current I with the beam current (I_{Beam}) and the area A with the size of the beam spot (A_{Beam}). The circular beam spot was treated as a squared profile (A_{Beam}) with side length equal FWHM of the Gaussian beam to simplify the calculations without losing accuracy. As the beam overlap was constantly set to 50%, each quadrant of these squared areas (A_{Beam}) is additionally irradiated during patterning by the surrounding 8 nearest neighboring patterning pixels. This finally leads to a factor of 4 for the total dose, explaining the multiplication term in equation (2). For the time t one can now set the pixel dwell time DT multiplied by the number of passes P. As alternative we may take the area spanned by the POP which is one fourth of the beam area. Note, we are aware that edge patterning points would require a different treatment with different multiplication factors. However, given the fact that a pattern with total area of 4 μm^2 yields between 5000 and 20000 points depending on the beam diameter, we treat edge patterning points as negligible.

$$D \cdot A = I \cdot t \tag{1}$$

$$D = \frac{I \cdot t}{A} = 4 \cdot \frac{I_{Beam} \cdot DT \cdot P}{A_{Beam}} = \frac{I_{Beam} \cdot DT \cdot P}{A_{POP}} \tag{2}$$

4 Modeling and fitting

The interaction of electrons with the TMSC and further its regenerated products may be described by chemical formalisms as depicted in equation (3). We have demonstrated that cellulose is an intermediate product by FTIR and indirect enzymatic degradation. However, we also observed the generation of non-degradable material with ongoing dwell-times (DTs) which most likely results from electron induced damage of the cellulose backbone. It is so far not clear, what products are generated precisely, but is feasible to assume that the main portion is carbon. We therefore may write the chemical reaction as follows:

$$TMSC + e^{-}_{acc} \rightleftharpoons^{k_{1+}}_{k_{1-}} cellulose + e^{-}_{acc} \rightleftharpoons^{k_{2+}}_{k_{2-}} Carbon \quad (3)$$

Here, e^{-}_{acc} is representative for the accelerated electrons which contribute to such reactions. At first, it is feasible to neglect k_{1-} and k_{2-} as these reactions are rather unlikely. From here on k_{1+} and k_{2+} will be denoted as k_1 and k_2, respectively. Each partial reaction, first to cellulose and from cellulose to carbonized products follows by principle second order kinetics. Now, assuming a constant flow of electrons which is the case, at least for the dwell-time, we obtain a situation which is well known as pseudo first order chemical reactions (see equation (4) with $B_{(t)}$ = constant). In fact, our analysis to unravel the regeneration from TMSC to cellulose and further to carbonized and non-degradable material represents the corresponding reaction kinetics. By finding appropriate equations for this behavior, we may be able to fit the functions and derive important parameters in addition to the optimal dose. Further it would prove our hypothesis derived in equation (3). The following formalism is a concise summary of the theoretical background behind the used model. In the following, for simplicity variables summarized in Table S 2 will be used.

Table S 2: Summary of the in equation (4) to (13) used variables.

Parameter	Variable	Description
[TMSC]	$A_{(t)}$	Concentration of TMSC
$[e^{-}_{acc}]$	$B_{(t)}$	Concentration of electron: $B_{(t)} = B$ = constant
[Cellulose]	$C_{(t)}$	Concentration of pure cellulose: $C_{(t)} = (A_0 - A_{(t)}) - D_{(t)}$
[Carbon]	$D_{(t)}$	Concentration of carbon rich, beam damaged material
[ND]	$\gamma_{(t)}$	Concentration of non-degradable material: $\gamma_{(t)} = A_{(t)} + D_{(t)}$

For the concentration of TMSC over time equation (4) is immanent:

$$-\frac{dA_{(t)}}{dt} = k_1 \cdot A_{(t)} \cdot B \tag{4}$$

Reorganization and integration yields:

$$A_{(t)} = A_0 \cdot e^{-k_1 \cdot B \cdot t} \tag{5}$$

As experimental curves denote the non-degradable material we must have a look at $C_{(t)}$ which is:

$$-\frac{dC_{(t)}}{dt} = k_2 \cdot C_{(t)} \cdot B - k_1 \cdot A_{(t)} \cdot B = k_2 \cdot C_{(t)} \cdot B - k_1 \cdot B \cdot A_0 \cdot e^{-k_1 \cdot B \cdot t} \tag{6}$$

If, we use the expression for $C_{(t)}$ as depicted in equation (6), we may write:

$$\frac{d\gamma_{(t)}}{dt} = k_2 \cdot A_0 \cdot B - k_2 \cdot \gamma_{(t)} \cdot B - k_1 \cdot B \cdot A_0 \cdot e^{-k_1 \cdot B \cdot t} \tag{7}$$

This is an ordinary linear differential equation of $\gamma_{(t)}$ and thus of the concentration of non-degradable material. A solution to equation (7) may easily be found by using a variation of parameters ansatz. Here the homogenous part of the equation may be easily derived in similarity to equation (4).

$$\frac{d\gamma_{(t)}}{dt} + k_2 \cdot \gamma_{(t)} \cdot B = 0 \quad \Rightarrow \gamma_{h(t)} = C_h \cdot e^{-k_2 \cdot B \cdot t} \tag{8}$$

A variation of constant ansatz now is:

$$\gamma_{p(t)} = C_{h(t)} \cdot e^{-k_2 \cdot B \cdot t} \tag{9}$$

Straightforward differentiation and substitution to equation (7) yields:

$$\begin{aligned} C_{h(t)} &= \int (k_2 \cdot A_0 \cdot B - k_1 \cdot A_0 \cdot B \cdot e^{-k_1 \cdot B \cdot t}) \cdot e^{k_2 \cdot B \cdot t} dt = \cdots \\ &= A_0 + A_0 \cdot \frac{k_1}{k_1 - k_2} e^{-k_1 \cdot B \cdot t} + C_p \cdot e^{-k_2 \cdot B \cdot t} \end{aligned} \tag{10}$$

With homogenous and particular solution we can now find a general solution to equation (7):

$$\gamma_{(t)} = \gamma_{h(t)} + \gamma_{p(t)} = A_0 + A_0 \cdot \frac{k_1}{k_1 - k_2} e^{-k_1 \cdot B \cdot t} + C_{p+h} \cdot e^{-k_2 \cdot B \cdot t}$$

To solve for C_{p+h} we now have to find a boundary condition of equation (7). Needless to say, the equation must satisfy

$$\gamma_{(0)} = A_0 = A_0 + A_0 \cdot \frac{k_1}{k_1 - k_2} + C_{p+h} \tag{11}$$

or

$$C_{p+h} = A_0 \cdot \frac{k_1}{k_2 - k_1} \tag{12}$$

The final solution to this problem is therefore as follows:

$$\gamma_{(t)} = A_0 + A_0 \cdot \frac{k_1}{k_1 - k_2} e^{-k_1 \cdot B \cdot t} + A_0 \cdot \frac{k_1}{k_2 - k_1} \cdot e^{-k_2 \cdot B \cdot t} \tag{13}$$

By substitution of the general terms in equation (13) we then get the fitting function with parameters a_1, a_2,b_1 and b_2.

$$[ND] = a_1 + a_2 \cdot \left(e^{-b_1 \cdot t} - e^{-b_2 \cdot t}\right) \tag{14}$$

Furthermore a_2 may be expressed by a_1, b_1 and b_2, which yields the final fitting function:

$$[ND] = a_1 + a_1 \cdot \frac{b_1}{b_1 - b_2} \left(e^{-b_1 \cdot t} - e^{-b_2 \cdot t}\right) \tag{15}$$

Data obtained by fitting equation (15) to experimental evaluated data is shown in Table 9 and Figure S 5 - Figure S 7.

Table S 3: Fit parameters obtained from equation (15) for each U_{Beam}, I_{Beam} pair with the corresponding point pitch (PP) used in the calculation of the current density J.

**U_{Beam}**	**I_{Beam}**	**PP**	**J**	**a1**	**Δa1**	**b1**	**Δb1**	**b2**	**Δb2**
kV	*pA*	*nm*	*pA/nm^2*						
2	2.5	7.4	0.34	1.01	0.01	0.19	0.01	0.14	0.15
2	13	8.1	1.60	0.94	0.02	0.50	0.03	0.17	0.13
2	53	10.4	5.10	0.95	0.02	1.17	0.05	0.30	0.02
2	210	16.6	12.65	1.06	0.03	1.88	0.08	0.41	0.02
5	5	4.0	1.25	0.98	0.02	0.36	0.02	0.27	0.02
5	25	5.0	5.05	1.02	0.01	1.26	0.03	0.73	0.03
5	98	7.5	13.15	1.05	0.01	1.89	0.06	0.99	0.04
5	400	13.6	29.52	1.05	0.01	2.45	0.10	1.06	0.05
10	7.5	2.7	2.78	0.93	0.01	0.68	0.02	0.52	0.02
10	33	3.6	9.17	1.07	0.01	2.02	0.06	0.79	0.03
10	130	5.8	22.41	1.06	0.01	2.87	0.05	0.92	0.02
10	540	11.0	49.09	1.10	0.01	3.61	0.10	1.07	0.03

Table S 4: Calculated values for b2/b1 and the corresponding optimal Dwelltimes (DT_{opt}) and Doses ($Dose_{opt}$).

**b2/b1**	**DT_{opt}**	**ΔDT_{opt}**	**$Dose_{opt}$**	**$\Delta Dose_{opt}$**
	µs	*µs*	*C/m^2*	*C/m^2*
0.71	6.11	7.64	0.28	0.35
0.33	3.29	0.39	0.65	0.08
0.25	1.57	0.07	0.77	0.03
0.22	1.04	0.08	0.79	0.06
0.75	3.24	1.81	1.01	0.57
0.58	1.03	0.21	1.05	0.22
0.52	0.72	0.07	1.27	0.13
0.43	0.60	0.05	1.31	0.11
0.77	1.68	1.50	1.72	1.55
0.39	0.76	0.08	1.94	0.21
0.32	0.58	0.02	2.25	0.08
0.30	0.48	0.02	2.14	0.07

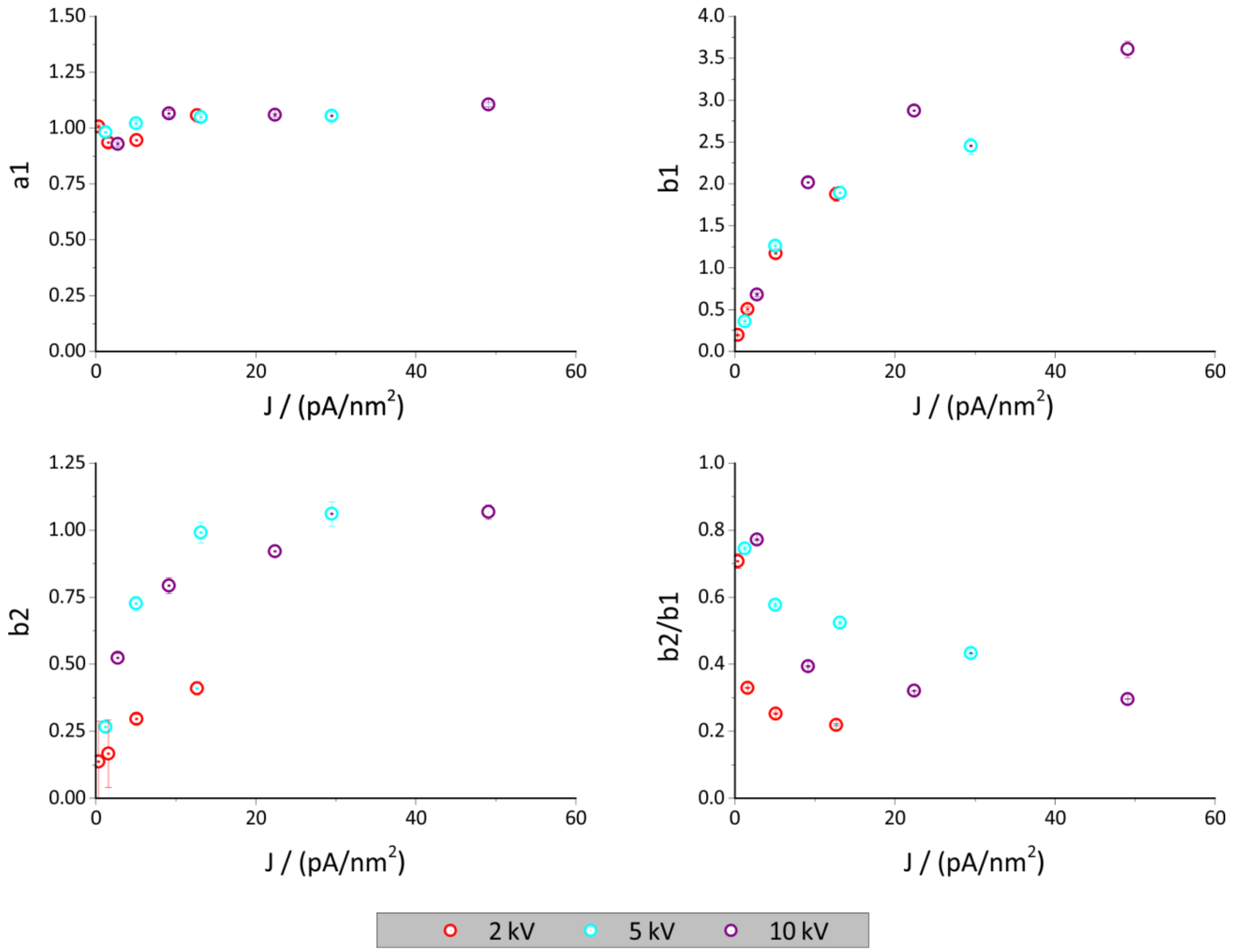

Figure S 4: Plotted values for a1, b1, b2 and the ratio of b2/b1 for different current densities J. Tabulated data can be seen in

Table S 3 and Table S 4.

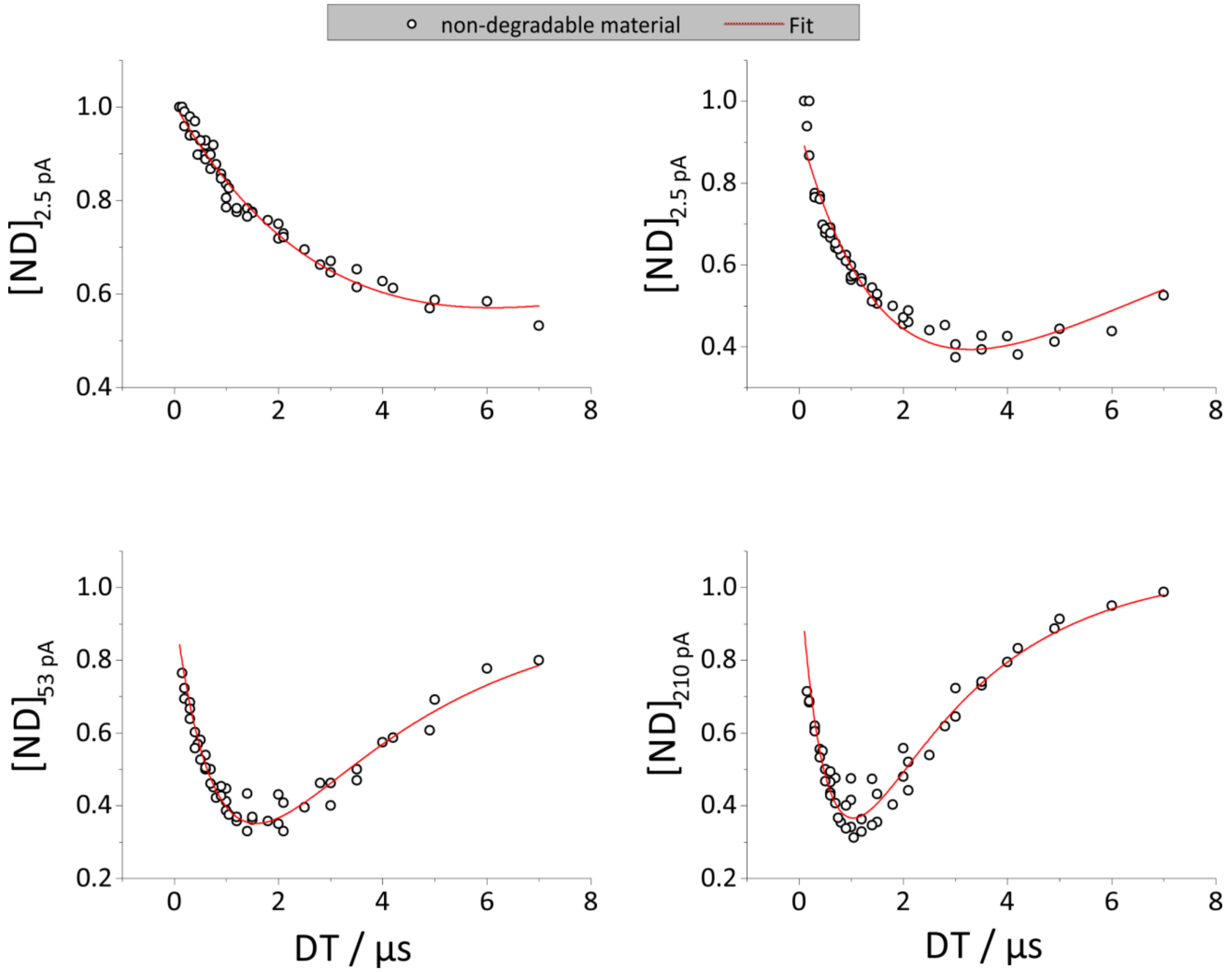

Figure S 5: Non-degradable material for 2 keV electrons evaluated from patterns as depicted in Figure S1. For the measurement height data before and after enzyme treatment is set into relation. Curves are fitted according to equation (14).

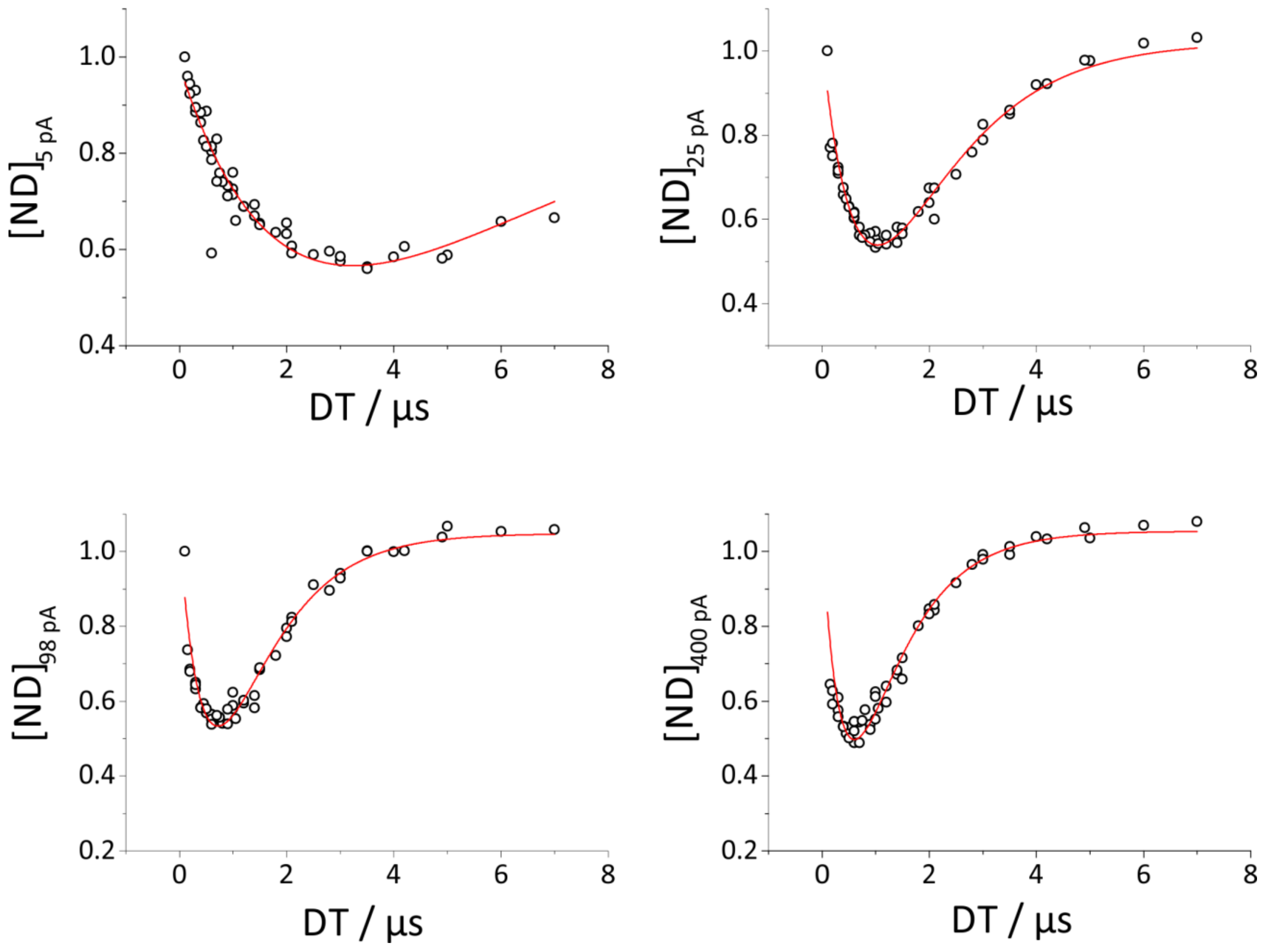

Figure S 6: Non-degradable material for 5 keV electrons evaluated from patterns as depicted in Figure S1. For the measurement height data before and after enzyme treatment is set into relation. Curves are fitted according to equation (14).

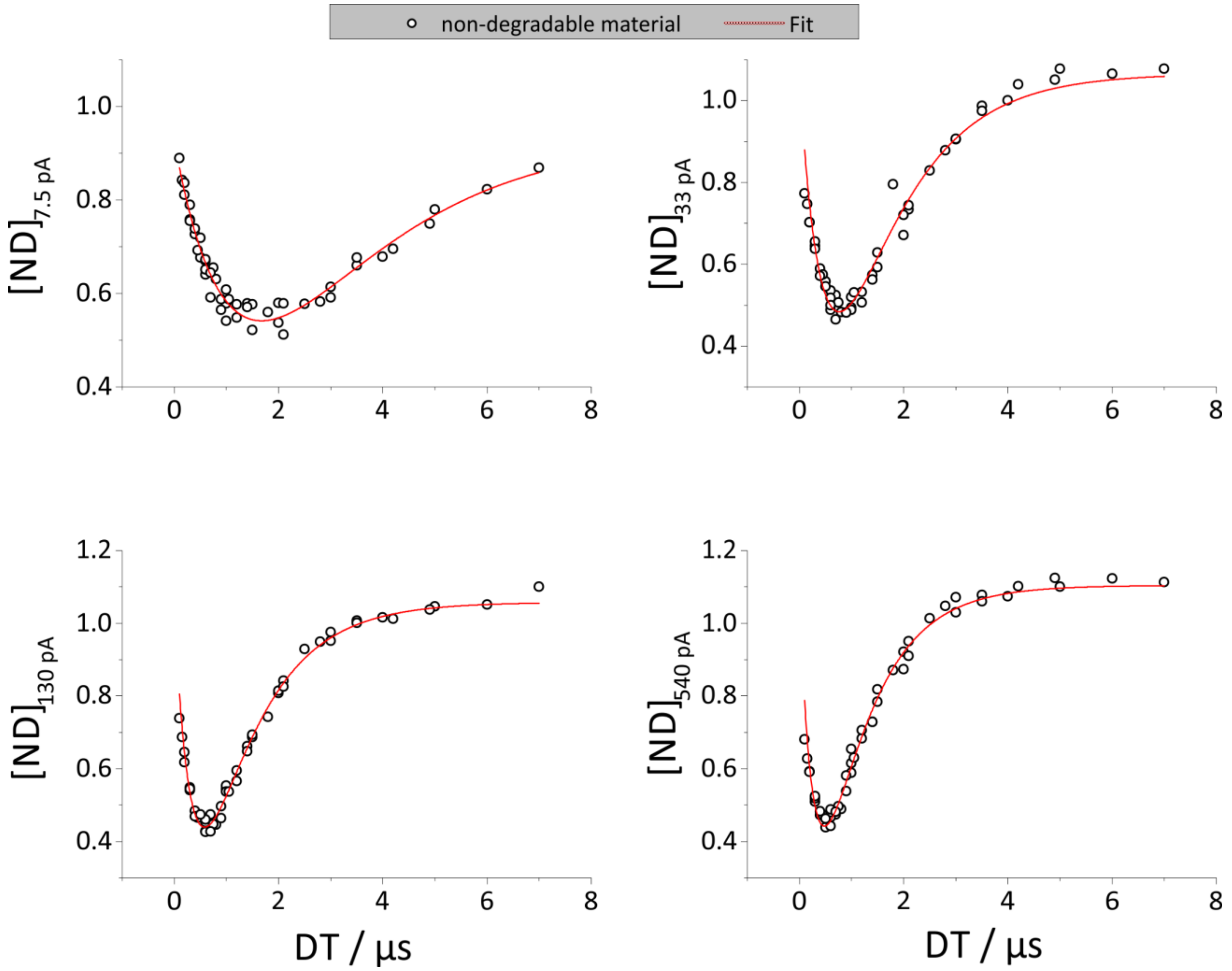

Figure S 7: Non-degradable material for 10 keV electrons evaluated from patterns as depicted in Figure S1. For the measurement height data before and after enzyme treatment is set into relation. Curves are fitted according to equation (14).

5 BSE Broadening

A more detailed investigation concerning broadening effects reveals two different influences which, however, are essential to achieve highest lateral resolution. For lowest primary electron energies of 2 keV a structure broadening effect in the range of 20 – 30 nm was found for ideal doses as representatively shown by AFM 3D height image in Figure S 8 (right image). For higher primary energies of 5 keV and 10 keV a stronger proximity broadening was found as evident from the AFM 3D height images at the center and at the right, respectively. According simulations concerning the BSE radius of the full TMSC / SiO_2 / Si stack (100 nm / 5 nm / bulk) reveal histograms as representatively shown bottom right in Figure S 8. Both, experimentally determined and simulation data have then been directly compared in the central graph of Figure S 8 by black triangles and the red colored background, respectively. The two dashed lines indicate a 95 % and 66 % decay of BSE intensity as shown in the bottom right inset. Now re-considering the simulation data in Figure 4 of the main manuscript it becomes clear that for the 2 keV situation, only a very small fraction of electrons reach the substrate. Therefore, the broadening effects for the respective 2 keV FEBIC structures are mainly determined by TMSC-related BSE contributions (further denoted as BSE_{TMSC}). In contrast, for 5 keV and 10 keV structures, a significant part of the electrons penetrate into the SiO_2 / Si substrate (see Figure 4) and cause a clear substrate related BSE proximity effect (denoted as BSE_{SUB}) aside the intended area as evident by the different appearance in the AFM 3D height images in Figure S 8. Therefore, we can state that all deposits reveal an intrinsic BSE_{TMSC} broadening which is responsible for the maximum achievable resolution in analogy to FEBID process[2]. Once the electrons reach the substrate, BSE_{SUB} contributions strongly reduce the achievable lateral resolution which demonstrates the need for careful adaption of the primary electron energy with respect to the TMSC layer thickness.

---

[2] Arnold, G.; Timilsina, R.; Fowlkes, J. D.; Orthacker, A.; Kothleitner, G.; Rack, P. D.; Plank, H. Fundamental Resolution Limits during Electron Induced Direct Write Synthesis. *ACS Appl. Mater. Interfaces* **2014**, *6*, 7380–7387

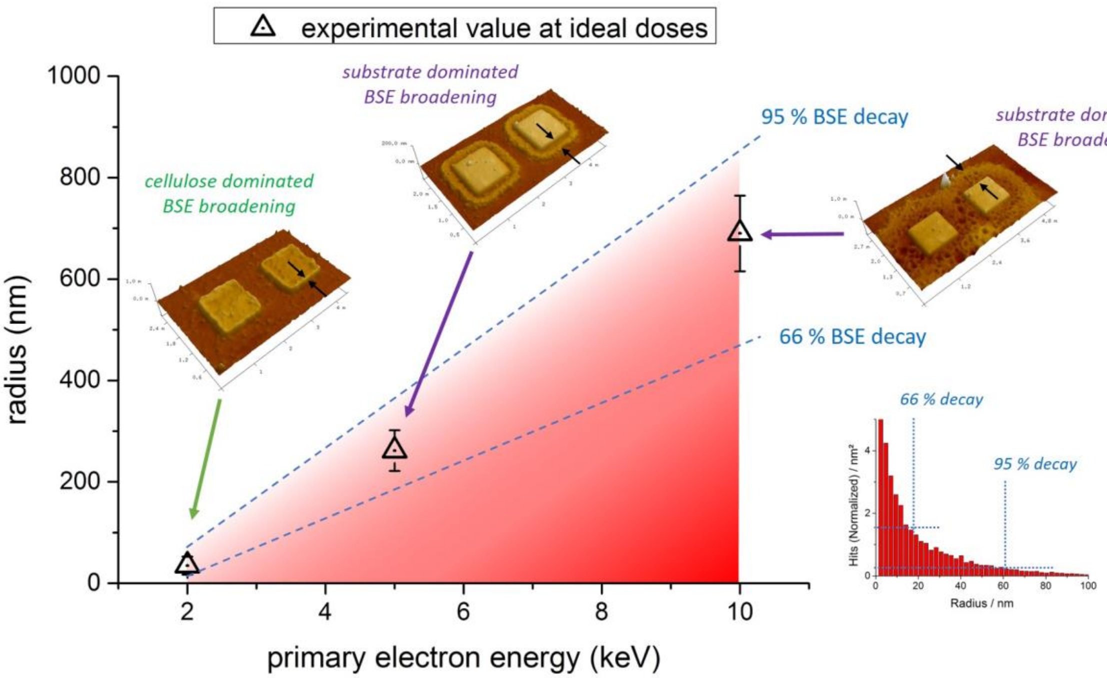

Figure S 8: AFM based identification of broadening effects during FEBIC processes shown by the black triangles in dependency on the primary electron energy. The 3D AFM height images show converted pads after removal of the unexposed TMSC. As evident, lowest primary energies show a small rim around the pads (left image) while higher energies of 5 keV and 10 keV result in clear proximity residue (center and right, respectively). The red area in the central graph gives the increasing BSE radius from the full TMSC / SiO_2 / Si stack while the 66 % and 95 % decays are indicated by the dashed lines. The latter are illustrated by the small graph bottom right which gives a BSE radius histogram with according decay indications.

6 Electron penetration depth

To achieve successful application of FEBIC structuring on TMSC films, film thickness has to be considered. As shown in Figure 4 for a 100 nm thick film, energy distribution as an indicator for electron penetration depth is strongly shifted into the layer and substrate with increasing primary beam energy. In the case of thicker films, this has to be considered carefully as choosing the primary beam energy to low may result in incomplete curing due to insufficient penetration depth. We therefore applied additional simulations using the Monte Carlo method and the CASINO package (CASINO 2.48; Build 2.4.8.1). Simulations were performed on 100, 250,500,750 and 1000 nm thick films with a 3 nm layer of SiO_2 and an underlying Si Substrate. In order to give a guide for scientists according primary beam energy and film height we have chosen the following strategy: Instead of providing an optimal beam energy we decided to provide a minimal acceleration voltage based on the CASINO simulations. In detail, optimal primary beam energies are not clearly definable as special applications may require different beam energies. It is for instance well known that at very high primary beam energies, proximity effects may be reduced while the regeneration time may be strongly increased due to reduced secondary electron cross section. On the other hand lower beam energies may provide a faster regeneration time. However, there is a limit at lower beam energies when electrons do not fully penetrate the film. Therefore minimal beam energies where chosen to fulfil the following requirement: The minimal beam energy in Table S 5 is chosen when the maximum penetration depth is 30 % deeper as the TMSC film thickness. For instance 1000 nm would require an approximate penetration depth of 1333 nm or 333nm into the SiO_2/Si substrate. It is, however, recommended to use higher beam energies as low beam energies show a stronger electron concentration gradient throughout the film.

Table S 5: Minimal beam energies in dependence of the TMSC layer thickness to ensure complete conversion of the film. The simulation was performed on 10^6 electrons using CASINO software.

**TMSC layer thickness**	**Minimum primary beam energy**	**Beam diameter**
nm	*kV*	*nm*
100	2	33.2
250	4	27.2
500	6	27.2
750	8	22
1000	10	22

7 FEBIC Structures

FEBIC structures may simply be generated by black and white bitmaps. Bitmaps are loaded into in-house written software (SIL-engine[3]) which generates the corresponding stream files. Stream files are loaded into the patterning engine of a FIB Nova 200 (FEI, The Netherlands) and patterned into the film.

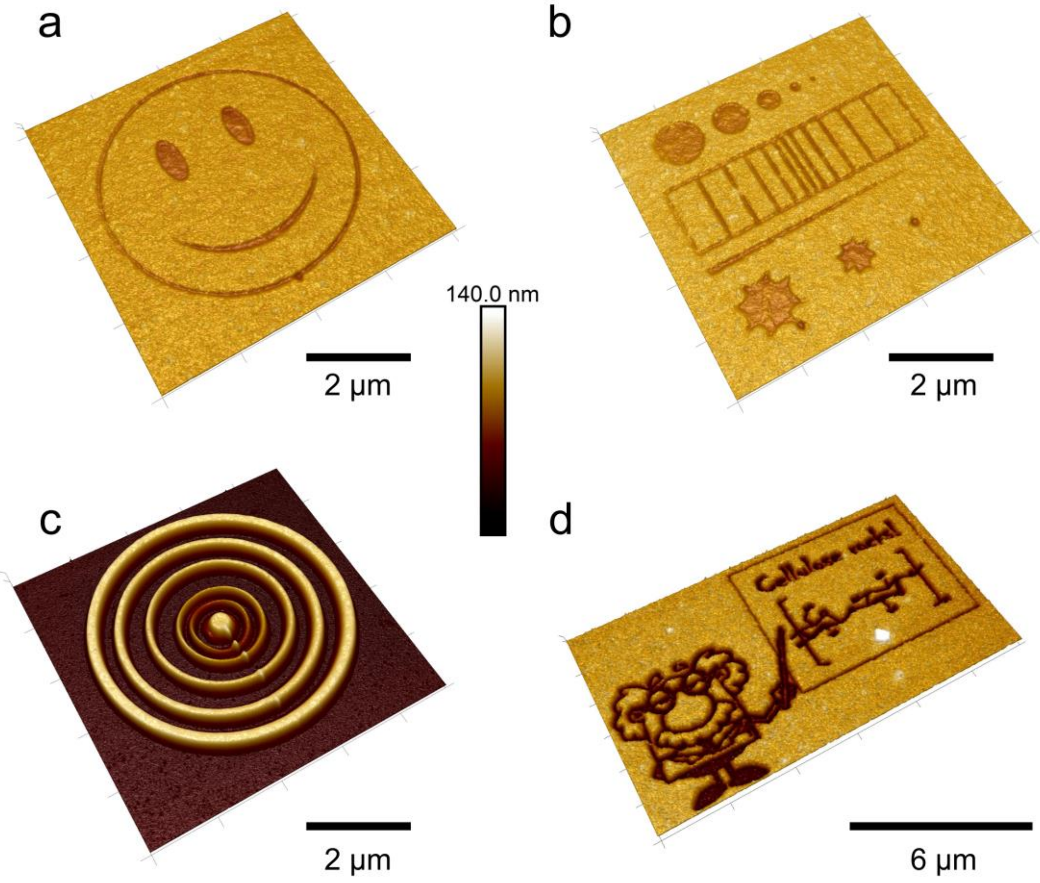

Figure S 9: 3D AFM images of representative FEBIC structures.(a) & (b) As patterned FEBIC structures of a smile and test-pattern. (c) Positive ring structure of cellulose, treaded after patterning in Xylol which removes the non-radiated TMSC. (d) Negative structure of post-enzyme treated FEBIC patterns revealing once more the cellulose nature of the regenerated pattern.

---

[3] Orthacker, R. Schmied, B. Chernev, J. E. Fröch, R. Winkler, J. Hobisch, G. Trimmel, H. Plank, Phys. Chem. Chem. Phys. 2014, 16, 1658.
